# Hémorragie cérébelleuse à distance après évacuation d'un hématome sous dural chronique par trou de trepan

**DOI:** 10.11604/pamj.2015.20.421.6365

**Published:** 2015-04-29

**Authors:** Abdelkarim Shimi, Nabil Elbakouri, Brahim Bechri, Ali Derkaoui, Mohamed Agouri, Mohammed Khatouf

**Affiliations:** 1 Service de Réanimation Polyvalente A1, CHU Hassan II, Faculté de Médecine et de Pharmacie, Université Sidi Mohamed Ben Abdellah, Fès, Maroc; 2 Service de Neurochirurgie, CHU Hassan II, Faculté de Médecine et de Pharmacie, Université Sidi Mohamed Ben Abdellah, Fès, Maroc

**Keywords:** Hémorragie cérébelleuse à distance, trou de trépan, hématome sous dural chronique, chirurgie supratentorielle, cerebellar hemorrhage, burr hole, chronic subdural hematoma, supratentorial surgery

## Abstract

L'hémorragie cérébelleuse à distance du site de la chirurgie ou remote cerebellar hemorrhage constitue une complication rare de la chirurgie intracranienne. la survenue d'une hémorragie cérébelleuse a distance dans les suites d'un drainage d'un hématome sous dural par trou de trépan reste un événement très rare. Nous rapportons le cas d'un patient ayant présenté ce syndrome après drainage d'un hématome sous dural chronique par trou de trépan avec évolution défavorable. Nous discutons à travers cette observation, les aspects diagnostics et étiopathogéniques de cette complication.

## Introduction

L'hémorragie cérébelleuse à distance du site opératoire(HCD) ou « remote cerebellar hemorrhage » est une complication classique de la chirurgie supratentorielle. La survenue d'un hémorragie cérébelleuse a distance dans le suites d'un drainage par trou de trepan d'un hématome sous dural chronique est un événement plus rare en neurochirurgie [1]. Nous rapportons le cas d'un patient âgé de 72 ans ayant présenté une hémorragie cérébelleuse dans les suites d'un drainage d'un hématome sous dural chronique par trou de trepan et nous discutons les mécanismes étiologiques possibles.

## Patient et observation

**Figure 1:**
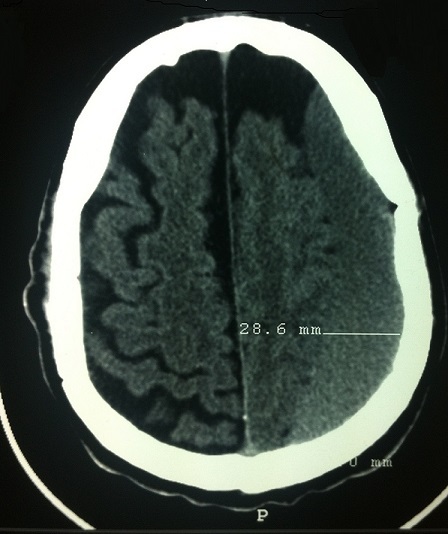
Hématome sous dural fronto-temporo-pariétal gauche

Patient de 72 ans, admis dans notre hôpital pour prise en charge d'un hématome sous dural chronique, dans ces antécédents on trouve un diabète de type II bien équilibré sous metformine. 10 jours avant son admission, le patient était victime d'un accident de la circulation sans retentissement neurologique, le scanner cérébral était demandé devant l'apparition de céphalée avec hémiparésie gauche. Le scanner cérébral a objectivé un hématome sous dural fronto-temporo-pariétal droit (Figure 1) de 28,6 mm, l'indication d’évacuation de l'HSDC par trou trépan sous anesthésie local était posée. Patient sans antécédents de troubles d'hémostase avec un bilan préopératoire correcte; le TP à 100%, les plaquettes à 225 000, hémoglobine à 13 g/dl. Durant la chirurgie la pression artérielle systolique maximale et minimale ont été respectivement de 115 et 137mmhg. La saturation pulsé en oxygène s'est situé entre 96 et 100% avec un débit d'oxygène en masque à 4 l/mn. Le geste a consisté à la réalisation d'un trou de trépan avec évacuation de l'hématome sous dural chronique et la mise en place d'un drain aspiratif qui a ramené 200ML sur 20 heures.

**Figure 2:**
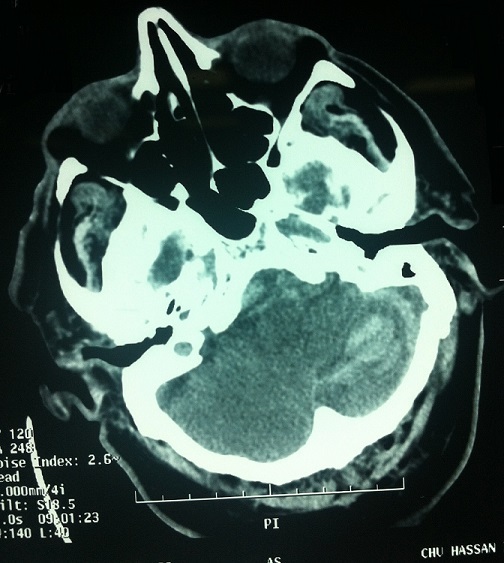
Hémorragie cérébelleuse gauche

L’évolution en post-opératoire était favorable avec récupération de déficit moteur et après un séjour de 4heures en salle de réveil le patient est transféré au service de neurochirurgie. Le lendemain matin, a un intervalle de 20heures du geste, le patient a présenté une crise convulsive tonico-clonique généralisée ayant cédée sous 10 mg de diazepam avec coma postcritique. Un scanner cérébral a été fait dans l'immédiat a objectivé la présence d'une hémorragie cérébelleuse a distance du site opératoire (Figure 2) sans indication d'une reprise chirurgicale. Le bilan biologique après le diagnostic de la complication était normal comme le bilan préopératoire. A J1 de son admission en réanimation, un électroencéphalogramme ne montrait aucune activité épilèptogène sous acide valproique. Le patient est décédé à J11 de son admission en réanimation dans un tableau de dysfonction multiviscéral dans les suites d'une pneumopathie nosocomiale à klebsiella pneumonie.

## Discussion

L'hémorragie cérébelleuse a distance (HCD) du site opératoire ou remonte cerebellar hemorrhage est définie par un saignement intraparenchymateux cérébelleux non contigu au site de l'intervention, il s'agit d'une complication rare de la chirurgie supratentorielle avec une incidence comprise entre 0,2 et 4,9% [2, 3]. Elle fait partie du cadre nosologique plus large des hémorragies intracérébrale à distance du site chirurgical. Cette complication est souvent asymptomatique est ignorée pour cette raison. Cette incidence est plus importante au décours du traitement des anévrysmes intracrâniens (3,5%) et après une lobectomie temporale (4,9%) [2, 3]. Une vingtaine de cas d'HCD survenant après chirurgie rachidienne ont été rapportés dans la littérature. La fréquence réelle de l'HCD est probablement sous estimé. lorsque l'HCD est symptomatique, la présentation clinique habituelle comporte céphalée, agitation et des troubles de la vigilance [3]. La survenue d'une hémorragie cérébelleuse a distance après drainage d'un hématome sous dural chronique par trou de trépan est rare, son incidence est estimé à 0, 14% [4]. L'hémorragie est donc fréquemment découverte au moment du réveil anesthésique soulignant l'importance de la surveillance en salle de surveillance post interventionnelle [5]. Néanmoins, il peut être porté à distance de la chirurgie, des cas à plus de 12 heures de celle-ci ayant été décrits [6]. Il est important de réaliser une imagerie cérébrale devant tout signe clinique anormal [7]. Comme dans notre observation le diagnostic est fait sur l'aspect scannographique au décours d'une complication chirurgicale précoce: troubles de la conscience, crise convulsives.

La TDM cérébrale révèle le plus souvent des signes des hyperdensités dans les sillons des hémisphères cérébelleux (signe de zèbre) et parfois des lésions hémorragiques intraparenchymateux principalement dans la partie supérieure de cervelet [8]. Le mécanisme de la survenue d'une hémorragie cérébelleuse a distance du site opératoire après drainage d'un hématome sous dural par trou de trépan reste inconnu. Mais il est supposé d’être multifactoriel [9]. L'hypertension artérielle per opératoire est considérés par certains auteurs comme un facteur de risque de survenue de cette complication [10, 11], pourtant la présence de l'hypertension artérielle peropératoire n'est rapporté dans la littérature que chez 22% des cas [9], dans notre cas, il y avait pas d'antécédents d'hypertension artérielle et les chiffres tensionnelles étaient correcte en per et en postopératoire. Les troubles de la coagulation sont ainsi considérés comme facteurs de risque prédisposant au saignement surtout en chirurgie supratentorielle [12]. Ce qui n'est pas le cas chez notre patient. Le bilan d'hémostase chez notre malade était correcte en pré et en postopératoire.

Le facteur causal selon l'hypothèse physiopathologique la plus étayée est une hypotension du liquide céphalorachidien [13]. Cette hypotension est responsable d'un affaissement des structures cérébrales (cervelet dans le cas de notre patient) cette affaissement entraine alors une traction sur les veines corticales sus tentorielle ou cerebelleuses. Cette distension peut conduire à la thrombose des plexus veineux cérébraux ou cérébelleux avec infarctus veineux ou à l'arrachement veineux avec hémorragie locale [14]. Le mécanisme est donc semblable à celui aboutissant aux hématomes cérébraux compliquant une ponction lombaire, cette dernière hypothèse peut expliquer le cas de notre patient, le drain a ramené 200 ml sur 20 heures peut être responsable d'une hypotension cérébrale conduisant à la survenue de cette complication.

Certains facteurs de risques sont débattus en fonction des auteurs: position peroperatoire (hyperextesion de la nuque, compression jugulaire interne entrainée par une déviation latérale de la tête, le décubitus ventral [15]. Par ailleurs, toutes les procédures qui aboutissent à une majoration de l'hypotonie cérébrale ou à une majoration du risque hémorragique doivent être considérées comme des facteurs de risque d'hémorragie cérébrale à distance et doivent donc les connaitre et de les éviter: perte de liquide céphalorachidien peropératoire importante; ouverture des citernes ou des ventricules; utilisation non pertinente du mannitol; coagulopathies; prise récente d'antiagrégants plaquettaire [14].

Lorsque le diagnostic d'hémorragie cérébelleuse à distance est effectué, le traitement est souvent symptomatique; retrait d'un drainage du liquide céphalorachidien, optimisation de la consommation cérébrale en oxygène (CMRO2) et maintien d'une pression de perfusion cérébrale correcte avec intubation, ventilation mécanique, sédation, antiépileptique, sans qu'il soit nécessaire en règle générale de recourir a une évacuation chirurgicale.

## Conclusion

L'hémorragie cérébelleuse à distance du site opératoire ou « remote cerebellar hemorrhage » est une complication rare de la neurochirurgie supratentorielle, elle doit être suspectée devant des complications postopératoire même lorsqu'il s'agit d’ une chirurgie mini-invasive comme le drainage par trou de trépan d’ un hématome sous dural chronique. Le diagnostic est affirmé par une imagerie cérébrale et la sa prévention passe par une déplétion contrôlée de LCR pendant l'intervention et le maintien d'une pression artérielle periopératoire normale. son traitement est souvent symptomatique permet une évolution le plus souvent favorable.
